# Cafedrine/theodrenaline in anaesthesia: Influencing factors in restoring arterial blood pressure

**DOI:** 10.1007/s00101-015-0005-y

**Published:** 2015-03-11

**Authors:** A. R. Heller, J. Heger, M. Gama de Abreu, M. P. Müller

**Affiliations:** Department of Anaesthesia and Intensive Care Medicine, Department of Anesthesiology and Critical Care Medicine, Medizinische Fakultät Carl Gustav Carus, Technische Universität Dresden, Fetscherstr. 74, 01307 Dresden, Germany

**Keywords:** Cafedrine/theodrenaline drug combination, Hypotension, Heart frequency, Beta-blocker effects, Heart failure, Cafedrin/Theodrenalin, Blutdruckabfall, Herzinsuffizienz, Betablocker-Effekte, Herzfrequenz

## Abstract

**Background:**

Hypotensive states that require fast stabilisation of blood pressure can occur during anaesthesia. In 1963, the 20:1 mixture of cafedrine/theodrenaline (Akrinor^®^) was introduced in Germany for use in anaesthesia and emergency medicine in the first-line management of hypotensive states. Though on the market for many years, few pharmacodynamic data are available on this combination net beta-mimetic agent.

**Aim:**

This study aimed to examine the drug combination in real-life clinical practice and recorded time to 10 % mean arterial blood pressure (MAP) increase and heart rate. Furthermore, potential factors that influence drug effectiveness under anaesthesia were assessed.

**Methods:**

Data were collected within a standardised anaesthesia protocol. A total of 353 consecutive patients (female/male = 149/204) who received cafedrine/theodrenaline after a drop in MAP ≥ 5 % were included in the study. The time to 10 % increase in MAP, dosage of cafedrine/theodrenaline, volume loading, blood pressure and heart rate were monitored over time.

**Results:**

Patients were a mean (standard deviation) of 64.4 ± 15.1 years old with a baseline MAP of 82 ± 14 mmHg, which dropped to a mean of 63 ± 10 mmHg during anaesthesia without gender differences. Cafedrine/theodrenaline (1.27 ± 1.0 mg/kg; 64 ± 50 µg/kg) significantly increased MAP (*p* < 0.001) by 11 ± 16 mmHg within 5 min, reaching peak values within 17.4 ± 9.0 min. Heart rate was not affected in a clinically significant manner. Cafedrine/theodrenaline induced a 10 % MAP increase after 7.2 ± 4.6 min (women) and after 8.6 ± 6.3 min (men) (*p* = 0.018). Independent of gender, the dose of cafedrine/theodrenaline required to achieve the observed MAP increase of 14 ± 16 mmHg at 15 min was significantly different in patients with heart failure [1.78 ± 1.67 mg/kg (cafedrine)/89.0 ± 83.5 µg/kg (theodrenaline)] compared with healthy patients [1.16 ± 0.77 mg/kg (cafedrine)/58.0 ± 38.5 µg/kg (theodrenaline)] (*p* = 0.005). Concomitant medication with beta-blocking agents significantly prolonged the time to 10 % MAP increase [9.0 ± 7.0 vs. 7.3 ± 4.3 min (*p* = 0.008)].

**Conclusion:**

Cafedrine/theodrenaline quickly restores MAP during anaesthesia. Female gender is associated with higher effectiveness, while heart failure and beta-blocker administration lower the anti-hypotonic effect. Prospective studies in defined patient populations are warranted to further characterise the effect of cafedrine/theodrenaline.

## Abstract (de)

**Hintergrund:**

Cafedrin/Theodrenalin (Akrinor^®^) wird in Deutschland für die Therapie von Blutdruckabfällen in Anästhesie und Notfallmedizin verwendet.

**Ziel der Arbeit (Fragestellung):**

Die Studie untersucht potentielle Faktoren, die die Wirksamkeit des Arzneimittels beeinflussen.

**Material und Methoden:**

Patientendaten wurden mittels Anästhesie-Protokollen gesammelt. 353 Patienten (weiblich/männlich = 149/204), die Cafedrin/Theodrenalin nach einem Abfall des mittleren arteriellen Blutdrucks (MAP) ≥ 5 % erhielten, wurden einbezogen.

**Ergebnisse:**

Die Patienten waren 64,4 ± 15,1 Jahre alt und hatten einen Ausgangs-MAP von 82 ± 14 mmHg, der während der Anästhesie unabhängig von Geschlecht auf einen Mittelwert von 63 ± 10 mmHg abfiel.

Cafedrin/Theodrenalin (1,27 ± 1,0 mg/kg/64 ± 50 µg/kg) erhöhte den MAP innerhalb von 5 min um 11 ± 16 mmHg (*p* < 0,001). Der maximale MAP war nach 17,4 ± 9,0 min erreicht; die Herzfrequenz wurde nicht im klinisch signifikanten Maß verändert. Cafedrin/Theodrenalin induzierte einen 10 % MAP-Anstieg nach 7,2 ± 4,6 min bei Frauen und nach 8,6 ± 6,3 min bei Männern (*p* = 0,018). Unabhängig vom Geschlecht waren die Dosen von Cafedrin/Theodrenalin, die zu dem beobachteten MAP-Anstieg um 14 ± 16 mmHg nach 15 min führten, signifikant unterschiedlich zwischen Patienten mit und ohne Herzinsuffizienz (1,78 ± 1,67 mg/kg (Cafedrin)/89,0 ± 83,5 µg/kg (Theodrenalin) vs. 1,16 ± 0,77 mg/kg (Cafedrin)/58,0 ± 38,5 µg/kg (Theodrenalin), *p* = 0,005).

Gleichzeitige Medikation mit Betablockern verlängerte die Zeit bis zum 10 % MAP-Anstieg [9,0 ± 7,0 vs. 7,3 ± 4,3 min (*p* = 0,008)].

**Schlussfolgerung:**

Theodrenalin/Cafedrin stellt den MAP nach Blutdruckabfällen schnell wieder her ohne die Herzfrequenz klinisch relevant zu verändern. Das Medikament zeigt eine höhere Wirksamkeit bei Frauen, während Herzinsuffizienz und Betablocker den Effekt schwächen.

Both general and regional anaesthesia are associated with a high rate of sympathicolysis-induced hypotension that requires fast-acting and reliable anti-hypotonic agents to stabilise blood pressure. For decades hypotension was avoided by the provision of excessive fluid preload, however, this practice faces increasing criticism [3]. In Germany, cafedrine/theodrenaline (Akrinor^®^) is approved as an anti-hypotensive agent alongside a few other agents.

## Background

Cafedrine/theodrenaline was introduced in Germany in 1963, and pre-clinical data showed a beta-adrenergic and an alpha-adrenergic component [22, 25, 26]. The net effect is beta-adrenergic [25]. The combination preparation increases mean arterial pressure (MAP), cardiac stroke volume and cardiac output [18, 25]. Compared to alpha-adrenergic sympathomimetic agents, cafedrine/theodrenaline was shown to cause less deterioration of renal, cerebral and coronary perfusion [5]. In addition, clinical practice with alpha-adrenergic sympathomimetic agents requires measures to control refectory critical bradycardia, for example with atropine [7]. A study in dogs demonstrated that increased myocardial oxygen consumption caused by positive inotropic cafedrine/theodrenaline effects are compensated for by enhanced coronary perfusion, increasing the myocardial oxygen supply [9]. The pharmacologic properties of this drug combination may explain the distinctly favourable effects in patients with chronic ischaemic heart disease [11, 15]. In addition, cafedrine/theodrenaline combination seems to offer benefits from a metabolic standpoint because of the lower lipolytic effect compared with catecholamines, particularly under hypoxic conditions in acute myocardial infarction or shock [21].

Although cafedrine/theodrenaline is widely accepted in the management of hypotensive states in anaesthesia, intensive care and emergency medicine [1, 6, 16], few pharmacodynamic data have been published [13], and it is therefore important to collect data under routine clinical practice conditions. Cafedrine/theodrenaline is routinely administered at our institution to patients in whom a quick and sustained MAP increase for 15–20 min is warranted and feasible with a single drug administration and without the vasopressor side effect of bradycardia; during these 15–20 min, fluid volume can be balanced. Gender-related differences in the ED_50_ of cafedrine/theodrenaline have previously been reported by our group [13]. To confirm and expand on this observation under routine anaesthesia conditions, the time to 10 % increase in MAP after administration of cafedrine/theodrenaline was examined. We hypothesized that gender, heart failure and use of beta-blockers influence the effectiveness of cafedrine/theodrenaline.

## Methods

This study was approved by the Institutional Review Board at the University Hospital Carl Gustav Carus Medical Faculty, Fetscherstrasse 74, Dresden, Germany (EK255122004, 17.01.2005). The records from 353 consecutive anaesthesia patients at our institution who received cafedrine/theodrenaline were collected and retrospectively evaluated. Patients were included in the study between July and November 2007, irrespective of the type of surgery, and received cafedrine/theodrenaline to manage hypotension during anaesthesia. To reflect a real-life cross-section of patients in a German hospital, patients from the following departments were included: orthopaedics, trauma surgery, neurosurgery, visceral- thoracic and vascular surgery, gynaecology, urology, oral and facial surgery and eye surgery.

All patients fasted 2 h prior to induction and received 7.5 mg midazolam OD (Dormicum^®^, Roche, Grenzach-Wyhlen, Germany) 45 min prior to arrival in the operating theatre. Concomitant medication, including anti-hypertensives such as beta-blockers, was continued as indicated by the standard operating procedures of the department. A 5-lead electrocardiogram, including measurement of segmental ST depression (II, aVF, V5), and pulse oximetry was recorded. Non-invasive oscillometric blood pressure was monitored in 5 min intervals on the right upper arm (IntelliVue^®^, MP70, Philips, Böblingen, Germany). Values were manually transferred to anaesthesia protocols. The following data were recorded: age, gender, height, body weight, current beta-blocker therapy, American Society of Anesthesiologists physical status and individual dosage of cafedrine/theodrenaline per injection.

General anaesthesia was induced with 1.5 mg/kg propofol (Propofol 1 %, Fresenius-Kabi, Bad Homburg, Germany) and 0.5 µg/kg sufentanil (Sufenta^®^ Janssen-Cilag, Neuss, Germany). Tracheal intubation was facilitated by 0.5 mg/kg rocuronium (Esmeron^®^, Organon, Oberschleißheim, Germany). General anaesthesia was maintained with desflurane (Suprane®, Baxter, Unterschleißheim, Germany) in O_2_/N_2_O (35 %/60 %). Patients were mechanically ventilated with a minute volume adequate to maintain end-tidal pCO_2_ of 36–40 mmHg at a fresh gas flow of 1 l/min (Primus^®^, Dräger, Lübeck, Germany). Neuraxial anaesthesia (spinal anaesthesia, epidural anaesthesia) was performed with approved local anaesthetics in awake seated patients [19].

In patients with ≥ 5 % drop in MAP cafedrine/theodrenaline was administered to maintain MAP within a 20 % drop from baseline. We chose the 5 % drop in MAP as the inclusion criterion to raise awareness that hypotension even in this range may be associated with late complications. Timing and doses were recorded in anaesthesia protocols. The observation period in this study was limited to 30 min after the drop in MAP because the variability of factors affecting MAP, such as additional volume therapy, ongoing blood loss and the use of other drugs increases with time. Individual dosage was defined in accordance with a previous dose-finding study, in which the ED_50_ of cafedrine to achieve a 10 % MAP increase within 10 min was 0.53 mg/kg [13].

### Administration of cafedrine/theodrenaline

An ampoule of 2 ml cafedrine/theodrenaline (Akrinor^®^, AWD.pharma GmbH & Co. KG, Dresden, Germany) contains 200 mg cafedrinhydrochloride, 10 mg theodrenalinehydrochloride, 0.4 mg sodiumdisulfite, ethanol 96 %, glycerol 85 %, sodium acetate 3 H_2_O, acetic acid 99 % and water. To enable fairly precise dosing, 2 ml of cafedrine/theodrenaline were diluted in 8 ml of saline to a total of 10 ml, as suggested in the prescribing information. Throughout the manuscript doses are given for the cafedrine component of the mixture. Due to the fixed milligram ratio of 1:20, the dosage of theodrenaline can be calculated by dividing the respective cafedrine dose by 20.

### Endpoints

The primary endpoint was time to 10 % increase in MAP after administration of cafedrine/theodrenaline. Other study endpoints were stability of heart frequency and time to onset of effect dose relative to gender, beta-blocking agents and the presence of heart failure (NYHA ≥ 1).

### Statistical analysis

Mean arterial blood pressure was calculated as MAP = diastolic + (systolic-diastolic)/3. All data were anonymised and analysed using the Statistical Package for Social Sciences for Windows, version 17.0 (SPSS, Inc., Chicago, IL, USA). The primary study hypothesis was tested using a Kaplan–Meier analysis followed by a log-rank test. General linear model statistics according to a two-way analysis of variance were applied for repeated measurement analyses, followed by Sidak alpha adjustment for multiple comparisons. A paired *t*-test served for analysing point to point changes in haemodynamics. To ensure equal distribution of risk factors between the observed cohorts, categorical variables were compared with χ^2^ statistics.

**Fig. 1:**
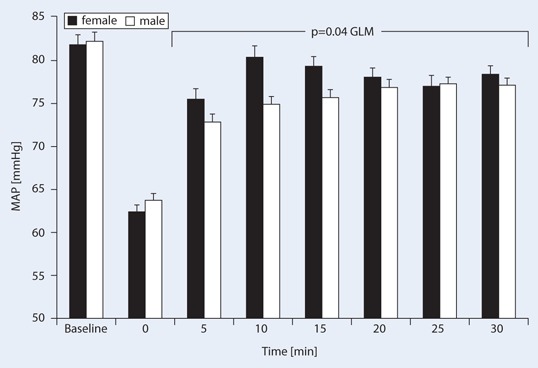
Effects of cafedrine/theodrenaline on mean arterial pressure (MAP, mean ± SE) injected at time point 0 (reference value) in male and female patients. Both MAP drop and increase were statistically significant (*p* < 0.001, paired *t*-test), as was the between-group comparison with general linear model (GLM) according to two-way analysis of variance (ANOVA) (*p* = 0.04)

**Fig. 2:**
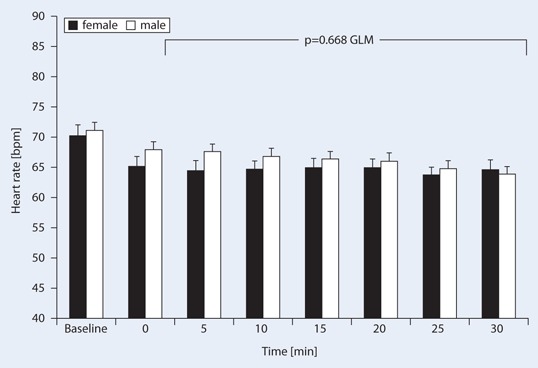
Effects of cafedrine/theodrenaline on heart rate (mean ± SE) injected at time point 0 (reference value) in male and female patients. The difference between genders was not significant (*p* = 0.668), while the heart rate difference over time was statistically significant (*p* < 0.001)

Data are expressed as mean ± SD. To support readability and interpretation in Figs. 1 and 2, data are presented as mean ± SE. A *p*-value of < 0.05 was considered statistically significant.

## Results

Data were collected for 353 consecutive anaesthesia patients (mean age 64.4 ± 15.1) at a single institution who received cafedrine/theodrenaline to treat hypotensive states during surgery between July and November 2007. Demographic characteristics, baseline values and treatment are presented in Table 1. 172 and 181 patients received general and neuraxial anaesthesia, respectively.

**Table 1:** Demographic baseline characteristics and treatment (mean ± SD)

	Women (*n* = 149)	Men (*n* = 204)	*p*-value
Age (years)	65.6 ± 16.6	63.6 ± 13.8	n.s.
Weight (kg)	69.6 ± 18.4	81.3 ± 15.4	0.001
ASA class	2.4 ± 0.7	2.5 ± 0.6	n.s.
Heart failure NYHA ≥ 1 [n (%)]	35 (23.5 %)	32 (15.7 %)	n.s.
Current beta-blocker therapy [n (%)]	50 (33.6 %)	61 (29.9 %)	n.s.
Heart failure NYHA ≥ 1 and beta-blocker therapy [n (%)]	18 (12.1 %)	18 (8.8 %)	n.s.
Score by Canadian Cardiovascular Society	0.2 ± 0.5	0.1 ± 0.4	n.s.
Volume load with crystalloids (ml)	481 ± 293	493 ± 287	n.s.
Baseline MAP (mmHg)	81.8 ± 12.8	82.2 ± 14.5	n.s.
MAP after decrease (mmHg); reference	62.4 ± 8.9	63.8 ± 11.4	n.s.
Dosage Akrinor^®^ (cafedrine mg/kg)^a^	1.3 ± 1.0	1.2 ± 1.0	n.s.

*ASA* American Society of Anesthesiologists, *NYHA* New York Heart Association, *MAP* mean arterial pressure

^a^Cafedrine:theodrenaline fixed milligram ratio = 20:1

The MAP dropped from a mean baseline value of 82 ± 14 to 63 ± 10 mmHg (equivalent to 78 ± 12 % of the baseline level; *p* < 0.001). MAP increase after 5 min was also statistically significant (*p* < 0.001), however mean baseline MAP values and the drop during surgery did not differ between genders (Fig. 1, Table 2). MAP increased by 11 ± 16 mmHg (*p* < 0.001) 5 min after administration of cafedrine/theodrenaline, while heart rates remained at a mean of 66 ± 18 bpm over 20 min. The highest MAP values were achieved 17.4 ± 9.0 min after the drop and administration of 1.27 ± 1.0 mg cafedrine/kg, independent of gender. For ease of transferring applied cafedrine/theodrenaline dosages into the reader’s clinical practice, Table 3 gives the relevant standard doses, dilutions and relations to body weight. Heart rates before the drop in MAP were a mean of 71 ± 18 and 67 ± 19 bpm after the drop (*p* < 0.001).

**Table 2:** Gender-related pharmacodynamic effects of cafedrine/theodrenaline (mean ± SD)

	Women (*n* = 149)	Men (*n* = 204)	*p*-value
10 % MAP increase (min)	7.2 ± 4.6	8.6 ± 6.3	0.018
Time to maximum MAP effect (min)	17.0 ± 9.1	17.7 ± 9.0	n.s.

**Table 3:** Conversion table for cafedrine/theodrenaline dosages into ml of standard dilutions

Akrinor^®^ ampoules	1	1/2	1/4
Cafedrine (mg)	200	100	50
Theodrenaline (mg)	10	5	2.5
Akrinor^®^ solution undiluted (ml)	2	1	0.5
Akrinor^®^ 1 ampoule diluted to 10 ml (ml)	10	5	2.5
Mean study dosage Akrinor^®^ per 75 kg BW (amp)^a^	0.48		

^a^Study mean dosage used was 1.3 ± 1.0 mg/kg (Cafedrine), 64 ± 50 µg/kg (Theodrenaline)

### Cafedrine/theodrenaline effectiveness according to gender

**Fig. 3:**
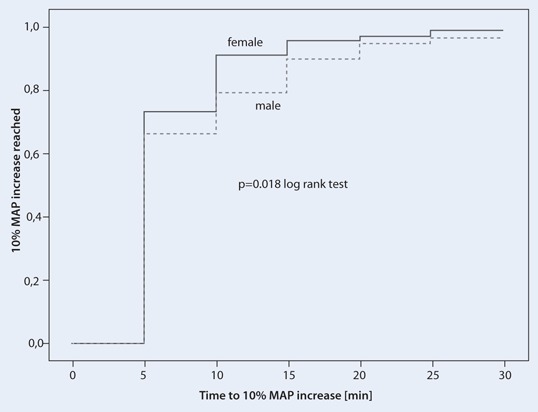
Kaplan–Meier analysis of the time to 10 % mean arterial pressure (MAP) increase in male (*dotted line*) and female (*solid line*) patients after cafedrine/theodrenaline injection at time point 0 (*p* = 0.018, log-rank test)

Heart rates did not differ significantly between genders (Fig. 2). After the drop in blood pressure cafedrine/theodrenaline induced a 10 % increase in MAP significantly earlier in women 7.2 ± 4.6 vs. 8.6 ± 6.3 min in men (*p* = 0.018) (Table 2, Fig. 3), however, the duration of the pressure-elevating effect as assessed by the time to peak MAP did not differ in a gender-related manner (Table 2).

### Cafedrine/theodrenaline in patients with heart failure

**Fig. 4:**
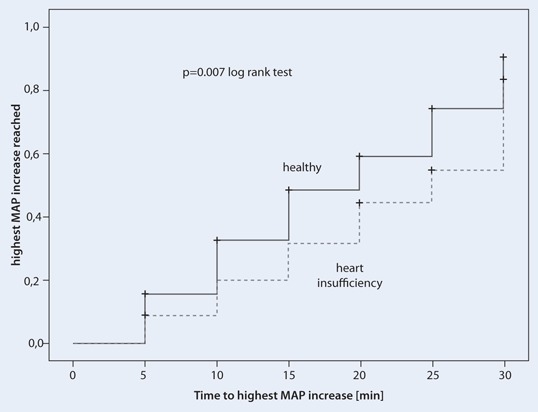
Kaplan–Meier analysis of the time to highest mean arterial pressure (MAP) after cafedrine/theodrenaline injection at time point 0 in patients with heart failure (NYHA ≥ 1, *dotted line*) and healthy patients (*solid line*) (*p* = 0.007, log-rank test). Cases not reaching a MAP increase of 20 % within 30 min were censored

The time to highest MAP was longer for the 67 patients with heart failure than for patients without heart problems (*p* = 0.007) (Fig. 4). In addition, the dose (mg/kg body weight) to achieve MAP increase in a similar range at 15 min (by 14 ± 16 mmHg in heart failure patients and by 14 ± 14 mmHg in healthy patients) were 1.78 ± 1.67 mg/kg in patients with heart failure and 1.16 ± 0.77 mg/kg in the healthy cohort (*p* = 0.005). This difference was independent of gender.

### Cafedrine/theodrenaline in patients with beta-blocking agents

**Fig. 5:**
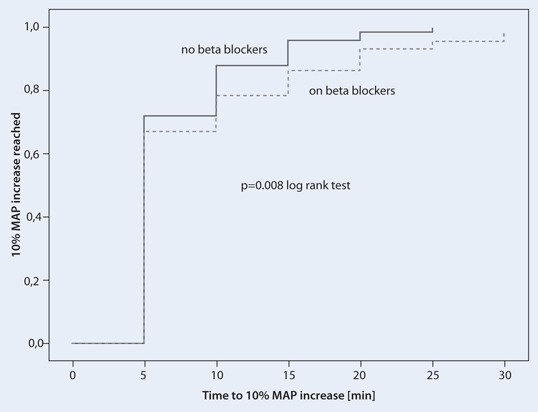
Kaplan–Meier analysis of the time to 10 % mean arterial pressure (MAP) increase after cafedrine/theodrenaline injection at time point 0 in patients with (*dotted line*) and without (*solid line*) current beta-blocker therapy (*p* = 0.008, log-rank test)

Concomitant medication with beta-blocking agents significantly prolonged the time to 10 % increase in MAP (*n* = 111, 9.0 ± 7.0 vs. 7.3 ± 4.3 min, *p* = 0.008) (Fig. 5). Factors potentially affecting the effectiveness of beta-stimulants such as the extent of volume load (492 ± 288 ml without beta-blocking agents and 475 ± 289 ml with beta-blocking agents) prior to blood pressure drop did not differ between the groups.

## Discussion

### Risks of hypotonia during anaesthesia

Induction of anaesthesia induces sympathicolysis, regardless of the type of anaesthesia, whether general or neuraxial, and poses a risk of hypotension, in particular when the administration of intravenous fluids is restricted [12, 17, 27]. The dilation of resistance and capacitance vessels in the blocked area, with a subsequent decrease of pressure in the systemic and pulmonary circulation, leads to a reduction in cardiac preload and after-load, posing a risk of cerebral or myocardial ischaemia as well as acute renal failure [10, 14]. The use of balanced anti-hypotonic agents such as cafedrine/theodrenaline may be beneficial when combined with gentle volume loading, especially in elderly patients [4, 28] and during more extensive surgical interventions [20]. A pilot study [13] suggested that male gender, heart failure and beta-blocking agents are factors that negatively influence the effectiveness of cafedrine/theodrenaline. Here we performed more in-depth analyses (Kaplan–Meier analyses) using an independent patient population to further elucidate the influences. We aimed to include a broad range of patients that had a ≥ 5 % blood pressure drop to represent a real-life population, regardless of underlying conditions. The range of the drop in blood pressure may vary in relation to the underlying disease; this study focuses on the therapy of the hypotensive states.

### Blood pressure restoration with pure alpha- or beta-adrenergic agents vs. cafedrine/theodrenaline

Due to the limited half-life, bolus administration of catecholamines has only a short-term circulatory effect [7]. Moreover, bolus injection of pure alpha-agonists cause an undesirable direct increase in peripheral resistance, resulting in additional energy-consuming wall tension in the myocardium [5] and bradycardia, necessitating additional pharmacologic treatment with atropine [7]. Alternatively, pure beta-mimetic drugs induce a delayed increase in blood pressure, due to initial vascular beta_2_-stimulation and the dependency on vascular filling; as a consequence, undesirable increases in heart rate are observed [18].

Here we show that the administration of cafedrine/theodrenaline significantly increased MAP in both genders without affecting the heart rate in a clinically significant manner. These observations are in line with experimental investigations [9, 11] and small clinical studies [13, 24].

### Affecting the effectiveness: gender, beta-blocking agents and heart failure

The time to 10 % MAP increase in this cohort was longer in men and in patients who had previously received beta-blocking agents. The more rapid increase in MAP in women may be attributable to the lower cafedrine/theodrenaline ED_50_ [13] and/or the higher intravascular fluid volume is observed in women [23] due to the higher level of endogenous oestrogen and resulting increase in preload. The downregulation of beta-receptors in older men [8] and oestrogen receptor-dependent effect that protects women from left ventricular hypertrophy and congestive heart failure [2] might represent contributing factors as well. Although potentially not relevant under day-to-day circumstances, during acute sympathicolysis-dependent hypotension these factors [2, 8] could have a clinical effect and at least partially explain the gender-related difference in the time to effect after cafedrine/theodrenaline administration. The effect of concomitant beta-blocker therapy on the effectiveness of cafedrine/theodrenaline is not surprising, given the predominantly beta-mimetic effects of this drug combination [24, 25] Patients with heart failure required longer to reach the maximum MAP and required higher doses of cafedrine/theodrenaline. Apparently patients with heart failure are not able to respond to stimulation by alpha- and beta-adrenergic agents [13].

### Limitations

This study has a number of limitations. The association between heart failure and the use of beta-blockers and the influence of health status and gender on the type of anaesthesia (general vs. regional) administered may have introduced sources of selection bias, especially in patients with heart failure receiving beta-blockers. Larger prospective and controlled studies with a more strictly defined and stratified patient populations are certainly warranted.

## Conclusion for clinical practice

- Cafedrine/theodrenaline results in a rapid haemodynamic effect on blood pressure without affecting heart rate to a clinically significant extent.
- Male gender, heart failure and the previous administration of beta-blocking agents negatively affect the effectiveness of cafedrine/theodrenaline.
- Prospective studies to characterise the effect of cafedrine/theodrenaline in defined patient populations are warranted.
